# Simultaneous Activation of Complement and Coagulation by MBL-Associated Serine Protease 2

**DOI:** 10.1371/journal.pone.0000623

**Published:** 2007-07-18

**Authors:** Anders Krarup, Russell Wallis, Julia S. Presanis, Péter Gál, Robert B. Sim

**Affiliations:** 1 MRC Immunochemistry Unit, Department of Biochemistry, University of Oxford, Oxford, United Kingdom; 2 Department of Infection, Immunity and Inflammation, University of Leicester, Leicester, United Kingdom; 3 Institute of Enzymology, Biological Research Center, Hungarian Academy of Sciences, Budapest, Hungary; Institut Pasteur Korea, Republic of Korea

## Abstract

The complement system is an important immune mechanism mediating both recognition and elimination of foreign bodies. The lectin pathway is one pathway of three by which the complement system is activated. The characteristic protease of this pathway is Mannan-binding lectin (MBL)-associated serine protease 2 (MASP2), which cleaves complement proteins C2 and C4. We present a novel and alternative role of MASP2 in the innate immune system. We have shown that MASP2 is capable of promoting fibrinogen turnover by cleavage of prothrombin, generating thrombin. By using a truncated active form of MASP2 as well as full-length MASP2 in complex with MBL, we have shown that the thrombin generated is active and can cleave both factor XIII and fibrinogen, forming cross-linked fibrin. To explore the biological significance of these findings we showed that fibrin was covalently bound on a bacterial surface to which MBL/MASP2 complexes were bound. These findings suggest that, as has been proposed for invertebrates, limited clotting may contribute to the innate immune response.

## Introduction

The immune system is composed of many recognition and effector mechanisms the primary role of which is to eliminate invading pathogens, altered host cells and macromolecules. The complement system is one such system, and it is composed mainly of plasma proteins of which several circulate as serine protease zymogens. These, when activated, catalyse downstream events of the complement system resulting in an inflammatory response, direct lysis and opsonization of microorganisms. The complement system can become activated by any of three pathways: the classical pathway relies on C1q binding, which enables the protease zymogen C1r to auto-activate and subsequently activate another protease zymogen C1s. The active C1s then cleaves C4 and C2 to trigger the downstream reaction [1]. The other two activation pathways are initiated by spontaneous hydrolysis of C3 (alternative pathway) or upon binding of mannan-binding lectin (MBL) or ficolins to sugars or N-acetylated groups on the surface of microorganisms (lectin pathway) [2]. Neither MBL nor ficolins possess enzyme activity themselves but rely on the MBL-associated serine proteases (MASPs)1, 2 and 3, with which they circulate in complexes [3]. When MBL, L-ficolin or H-ficolin bind to a bacterial surface, the MASPs, which are homologues of C1r and C1s, become activated. Of the three MASPs only MASP2 is capable of activating complement by cleavage of C4 and C2. [4].

The coagulation system is another protein cascade of which most components circulate in plasma. The main role of the coagulation system is not neutralization of invading pathogens but to maintain the integrity of the circulatory system upon injury. The coagulation system can be activated by two pathways, the intrinsic and the extrinsic, of which both lead to the formation of the prothrombinase complex on phospholipid membranes. The prothrombinase complex is made up of factor Va and factor Xa and generates active thrombin by factor Xa-mediated double cleavage of prothrombin [5]. The generation of thrombin is regarded as the critical step in the coagulation cascade since thrombin mediates the functions that leads to the formation of blood clots by cleavage of fibrinogen and factor XIII and activation of platelets [6].

The complement and the coagulation systems have many similarities since both cascades utilize as catalysts multi-domain serine proteases with similar domain structure. The similarity of the two systems on the structural level can be demonstrated with phylogenetic analysis in which the proteases of the classical and the lectin pathways become grouped together with coagulation enzymes [7]. Both cascade systems are tightly regulated whereby the active enzymes have a short half-life but this is overcome by end-product amplification that is characteristic for protein cascades. The coagulation system serine proteases are regulated by serpins and secondarily by α_2_-Macroglobulin [8]. The C1r, C1s, MASP1 and MASP2 proteases are also regulated by a serpin (C1-inhibitor) but MASP1 and MASP2 regulation is more similar to that of coagulation proteases, since both also are inhibited by anti-thrombin III and α_2_-Macroglobulin [9], [10].

The similarities generally suggest that the proteases of the two protein cascades have originated from the same ancestral proteases. Here we have investigated a possible link on the functional level between the two systems since we have demonstrated that MASP2 is capable of activating prothrombin in a similar manner to factor Xa. Furthermore the MASP2-generated thrombin is able to cleave synthetic tripeptide substrates and activate two of its main protein substrates fibrinogen and factor XIII. This activity was identifiable both in fluid-phase by using a truncated rMASP2 construct (trMASP2) and a full-length rMASP2 bound to MBL. These findings indicate a possible physiological role for fibrinogen in the innate immune system of vertebrates, perhaps analogous to the suggested roles of coagulation in defense of invertebrates against infection [11].

## Results

**Figure 1:**
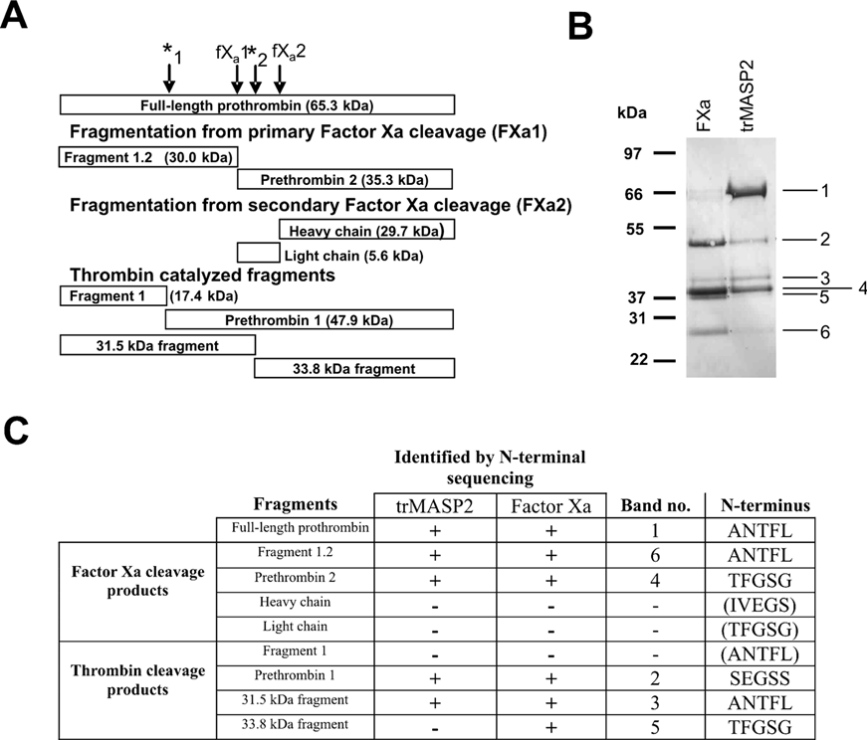
Prothrombin fragmentation. A) Prothrombin fragments generated during activation by factor Xa and cleavage by thrombin as would be observed on SDS-PAGE under reducing conditions (based on ref (5)). The arrows indicate the cleavage sites for factor Xa (fXa1 Arg273-Thr274 and fXa2 Arg322-Ile323) and thrombin (*1 Arg155-Ser156 and *2 Arg286-Thr287). The molecular mass of the fragments shown was calculated based on the primary sequence (Swiss-Prot entry P08709). B) The observed cleavage pattern of prothrombin incubated with factor Xa (FXa) (lane 1) or trMASP2 (lane 2) for 4 h at 37°C and analyzed by reducing SDS-PAGE and Coomassie Blue staining. The bands were identified by N-terminal sequencing together with theoretical and observed size. The 33.8 kDa fragment migrates faster than the 31.5 kDa fragment due to differences in the number of N-linked glycosylations. C) The sites of cleavage by factor Xa and trMASP2 are identical as confirmed by N-terminal sequencing as described in the text.

When prothrombin activation by factor Xa is analyzed by SDS-PAGE a complex fragmentation pattern is observed, since both factor Xa and the generated thrombin can each cleave prothrombin twice [5]. A schematic overview of the possible prothrombin fragments generated by factor Xa and thrombin is shown in fig 1A. In fig 1B we show SDS-PAGE analysis of the fragmentation of prothrombin when incubated with factor Xa or trMASP2. Identification of the bands was carried out by N-terminal sequencing and by comparing the observed molecular masses determined by their migration in SDS-PAGE gels to the calculated masses based on the primary sequence. Factor Xa and trMASP2 both generate the same cleavage fragments although in different quantities. Fragment 1.2 and prethrombin 2 is seen in both gel tracks, indicating that factor Xa and trMASP2 cleave at the fXa1 site (Arg^273^-Thr^274^). This was confirmed by N-terminal sequencing of the prethrombin 2 fragment formed by trMASP2 (fig 1C). The second factor Xa cleavage (fXa2, Arg^322^-Ile^323^) is not visible with either factor Xa or trMASP2 (fig 1B) perhaps because the heavy chain coruns with fragment 1.2, and the light chain is too small to be observed on the gel. However the fXa2 cleavage, which generates active thrombin, must occur with both factor Xa and trMASP2, as products of both the thrombin-mediated cleavages *_1_ and *_2_ are seen in fig 1B. These products are (*_1_) prethrombin 1 (fragment 1 has migrated off the gel) and (*_2_) the 31.5 and 33.8 kDa fragments. The N-termini of all the fragments visible on figure 1B were confirmed (fig 1C) except for the 33.8 kDa fragment in the trMASP2 lane (quantity too low). We were unable to detect all the products but this is due to lack of sensitivity of our detection system rather than them not being generated. Activation of prothrombin is a very rare proteolytic specificity. Apart from tryptase, the BRENDA enzyme database (http://www.brenda.uni-koeln.de/) records no other mammalian protease which does this. In contrast to trMASP2, truncated rMASP1 (trMASP1) does not cleave prothrombin (not shown).

**Figure 2:**
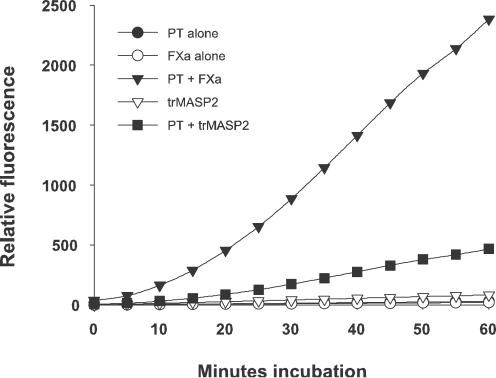
Thrombin activity generated by factor Xa and trMASP2 measured by cleavage of VPR-AMC. Samples (100 µl) contained 10 µg prothrombin (PT)+25 ng factor Xa (fXa), 25 ng factor Xa alone, 10 µg prothrombin+100 ng trMASP2, 100 ng trMASP2 alone or 10 µg prothrombin alone. As can be observed the cleavage of the substrate by factor Xa, trMASP2 and prothrombin is negligible, but incubation of prothrombin with factor Xa or trMASP2 generates activity. The figure shows the development of the relative fluorescence at different time points. A typical result from 3 experiments is shown.

To investigate to what extent thrombin generation proceeds we incubated factor Xa and trMASP2 with prothrombin, then measured the thrombin activity using a tripeptide substrate VPR-AMC which is selective for thrombin. The results are displayed in figure 2. Factor Xa alone, trMASP2 alone or prothrombin alone turn over the substrate to a negligible extent. However when factor Xa or trMASP2 are incubated with prothrombin, there is increased substrate turnover, consistent with generation of thrombin. Thrombin generation was further confirmed by studying fibrinogen turnover (see below). In the experiment shown in fig 2 prothrombin concentration is 100 µg/ml (approximately physiological). trMASP2 (100 ng) generates at each point about 20% as much thrombin activity as 25 ng factor Xa. Therefore, we assume that 25 ng trMASP2 would generate only 4–5% as much thrombin as 25 ng factor Xa. In fig 2, the curves are non-linear, as more thrombin is being generated with time. This leads to upward curvature. At later times substrate depletion and the instability of all 3 proteases leads to lower rates of substrate turnover. The observed reaction measures only production of active thrombin by 2 cleavages of prothrombin by factor Xa or trMASP2. However the competing reaction, 2 cleavages by thrombin to produce inactive products, is not measured directly but contributes to substrate depletion.

Factor Xa alone, however is not the major physiological activator of prothrombin. This is carried out by the factor Xa-factor Va prothrombinase complex which is the major activator during coagulation.

**Figure 3:**
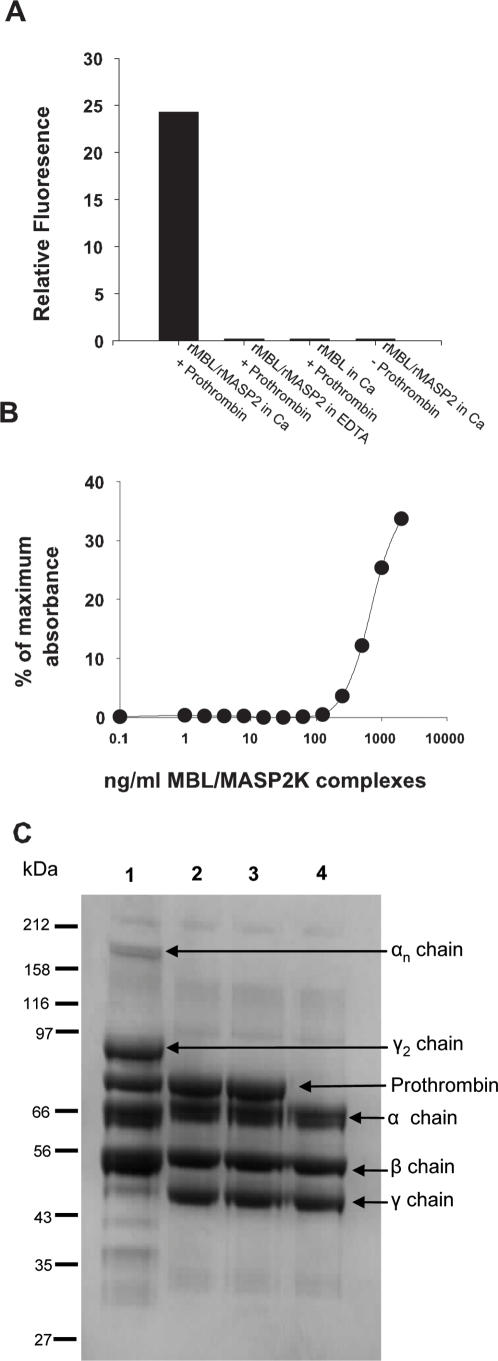
VPR-AMC cleavage (A), fibrin aggregation (B) and fibrinogen cleavage and cross-linking (C) by thrombin generated by mannan-bound MBL/MASP2K. In fig 3A, thrombin generation is shown by cleavage of VPR-AMC. Column 1 shows the VPR-AMC turn-over when MBL/MASP2K was incubated with mannan in the presence of Ca^2+^ ions, followed by addition of prothrombin. Column 2 is the control for column 1 where MBL/MASP2K complexes were bound in the presence of EDTA. Column 3 is a further control with no rMASP2 added, and Column 4 is the same as column 1, but without prothrombin. The figure shows the cleavage of VPR-AMC by thrombin (as relative fluorescence) after 4h at 37°C. Fig 3B shows the MBL/MASP2K dose-dependent fibrin polymerization in a microtiter well after incubation with prothrombin and fibrinogen for 3.5 hours. The X-axis shows a 2-fold dilution series of the MBL/MASP2K complexes starting at 200 ng/well. The background was defined as the activity from a sample in which MBL/MASP2K complexes were prevented from binding by the presence of EDTA and subtracted. Fig 3C shows generation of fibrinogen γ chain dimer and α chain oligomers. Sample numbers correspond to those in fig 3A. Only in lane 1 have the γ chain dimer as well as α chain oligomers been formed. This arises from fibrinogen activation followed by factor XIIIa mediated cross-linking. This occurs only in the presence of activated MBL/MASP2K complexes bound to mannan, and in the presence of prothrombin. A typical result from 3 experiments is shown.

Results in fig 2 were obtained with a truncated version of the MASP2 enzyme produced in *E.coli*, which lacks the CUB1-EGF-CUB2 domains which interact with MBL. We wanted to investigate if full-length MASP2 in complex with MBL also is capable of activating prothrombin when bound to a surface. To do this we used rat rMBL-A [12] and rat rMASP2K [13] using mannan as an activating surface. On mannan-coated microtiter plate wells MBL/MASP2K complexes were bound and incubated with prothrombin and VPR-AMC or fibrinogen. The results from these experiments can be seen in figure 3A and B, respectively. Figure 3A shows that only MBL/MASP2K complexes bound in the presence of Ca^2+^ ions and incubated with prothrombin generate active thrombin. All of the negative controls, i.e. MBL/MASP2K incubated with mannan in the presence of EDTA (where the complexes will not form and MASP2K is not activated), the controls with no MASP2K or with no prothrombin show no ability to cleave the substrate above background level (fig 3A columns 2, 3 and 4).

Figure 3B shows how the turbidity of a fibrinogen solution changes due to fibrin polymerization. A series of dilutions of MBL/MASP2K were bound in mannan-coated wells and subsequently incubated with prothrombin and fibrinogen. As can be observed the higher concentrations of rMBL/rMASP2K lead to polymerization and increase of light scatter at 405 nm showing the ongoing polymerization. The reaction has not reached its endpoint since the turbidity only is 35–40% of the value seen when active thrombin was directly added to the fibrinogen. Figure 3C shows SDS-PAGE analysis of fibrinogen following incubation in the mannan-coated wells with rMBL/MASP2K. Fibrinogen is a hexamer composed of 2 α, 2 β and 2 γ chains. Upon activation by thrombin the α and β chains are cleaved and the fibrinopeptides A and B are released. At the same time thrombin cleaves factor XIII generating factor XIIIa which is a transglutaminase capable of stabilizing fibrin clots by covalent linking of two γ-chains or multiple α-chains forming the γ_2_ and the α_n_ oligomer [14]. It was not necessary to add any factor XIII to the experiments since it is found in all fibrinogen preparations as a contaminant and its transglutaminase activity, i.e. the generation of the γ_2_ and multiple α_n_ oligomer, was used as a marker for early stage clot formation. Cleavage of the α and β chains and formation of γ_2_ and α_n_ clearly occurs in lane 1 which represents the MBL/MASP2K complexes bound in the presence of Ca^2+^ ions and incubated with prothrombin and fibrinogen. In the control lanes where MBL/MASP2K complexes were incubated in the presence of EDTA, or in the absence of MASP2K or prothrombin (lanes 2, 3 and 4) no fibrinogen cleavage or cross-linking can be observed. These experiments confirm that the MBL/MASP2K can generate functional thrombin, detected by activation of factor XIII and fibrinogen, which are two of the major protein substrates of thrombin.

**Figure 4:**
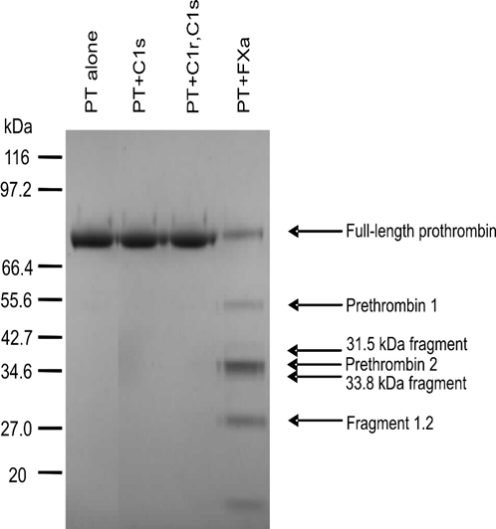
Reducing SDS-PAGE of prothrombin incubated at 37°C for 4 hours alone or with activated C1s, activated C1r,C1s mixture or factor Xa. Neither C1s alone or C1r, C1s mixtures are capable of cleaving prothrombin. Factor Xa is included as a positive control.

The specificity of the thrombin activation by MASP2 was examined in 2 ways. Firstly homologues of MASP2 namely C1r and C1s (and trMASP1, see above) were tested for their capacity to generate thrombin. Secondly trMASP2 was incubated with other protease proenzymes to see if active proteases were generated. We incubated prothrombin alone or with activated C1s or a mixture of activated C1r and C1s. Factor Xa was the positive control. Prothrombin activation was observed by analyzing cleavage patterns on reducing SDS-PAGE. As can be seen in figure 4 C1r and C1s do not cleave prothrombin. This shows that the ability of MASP2 to activate prothrombin is relatively specific. Additional experiments performed with other plasma protease proenzymes, prekallikrein and plasminogen showed that neither trMASP1 nor trMASP2 significantly cleaves prekallikrein during 1 h or 16 h incubation. Neither protease cleaves plasminogen to form the heavy and light chains of plasmin within 16 h at 37°C, as assessed by SDS-PAGE analysis (not shown). Incubation does not lead to the generation of the active proteases within 2 h at 37°C when investigated by using specific plasmin and kallikrein tripeptide substrates (not shown). These results confirm that the MASP2-mediated activation of prothrombin is relatively specific and is unique for C4 and C2 cleaving enzymes.

**Figure 5:**
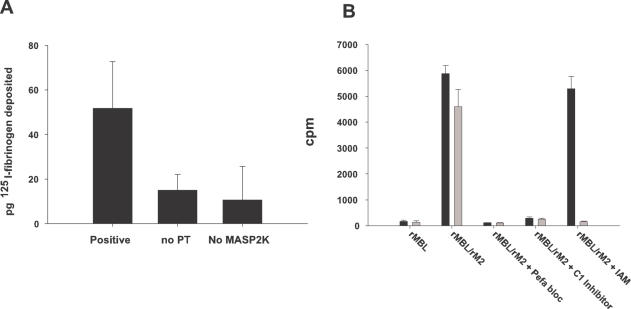
Fibrin deposition on MBL target surface. Fig 5A shows ^125^I-fibrin deposition on a mannan/fibrinogen coated surface to which rMBL/rMASP2K complexes are bound. The background (a control in which rMBL/rMASP2K complexes were prevented from binding to the surface due to the presence of EDTA) has been subtracted from the remaining samples. The figure shows that deposition above background level only occurs if rMASP2K is present and if the incubation with fibrinogen is in the presence of prothrombin. Incubation was for 4h at 37°C. Fig 5B ^125^I-fibrin deposition on *S. aureus*-derivatized beads. *S. aureus*-derivatized beads were incubated at 4°C with rMBL/rMASP2 complexes and subsequently with prothrombin and ^125^I-fibrinogen for 7 h at 37°C. The figure shows that fibrin gets deposited on the beads upon activation of prothrombin by rMBL/rMASP2 complexes (columns 2 and 5, closed bars). The fibrin bound to the surface also gets covalently cross-linked to the bacteria since it was not removed by washing the beads with urea (column 2, open bar). If the deposition was done in the presence of iodoacetamide (IAM), which inhibits factor XIIIa, the radioactivity associated with the beads was reduced to background level upon urea extraction (column 5, open bar). In the controls in which no rMASP2 was added or in the presence of protease inhibitors (C1 inhibitor or Pefabloc SC) no deposition on the beads could be observed. Each sample was tested twice and the error bars represent one standard deviation from the mean.

Since MASP2 circulates in complex with MBL and the ficolins and these have to bind to the surface of targets (eg bacteria) before MASP2 becomes activated, it was of interest to see if binding of the MBL/MASP2K complexes would result in fibrin deposition on the surface upon which the complex is bound. To investigate this two experiments were designed. In the first (fig 5A) we incubated MBL/MASP2K complexes on a fibrinogen/mannan coated plate and measured the deposition of ^125^I-fibrinogen in the wells after prothrombin activation. The column labeled positive is the sample in which MBL/MASP2K complexes were bound in the presence of Ca^2+^ ions and subsequently incubated with ^125^I-fibrinogen and prothrombin. The remaining two controls are either without MASP2K or without prothrombin. As can be observed the prothrombin activation by MASP2K leads to ^125^I-fibrin deposition in the wells showing that target surfaces indeed do get fibrin deposited. In this type of experiment no ^125^I-fibrin binding was found if the plate was coated with only mannan. However if a mixture of mannan and a protein (fibrinogen, β-casein or thiolester-cleaved α2M) was used, ^125^I-fibrin was bound. This is consistent with the need for glutamine or lysine side chains on the surface to which factor XIIIa can mediate covalent linkage. To investigate the deposition of fibrin further we derivatized beads with a non-encapsulated *S. aureus* strain (Wood), previously shown to be capable of binding MBL, to assess if fibrin deposition also happens on microorganisms [15]. MBL/MASP2K complexes were then bound to these beads, and prothrombin and ^125^I-fibrinogen added. After incubation the beads were washed and the associated radioactivity was measured. Column 2 and 5 on figure 5B show that only the beads incubated with MBL/MASP2K complexes get ^125^I-fibrin deposited on the surface. In the presence of iodoacetamide (IAM) however, the deposited fibrin could be removed by incubation with urea (Column 5, open bar) while this was not possible in the sample without IAM (Column 2, open bar). This is because IAM is an inhibitor of factor XIIIa, which is responsible for covalent cross-linking of fibrin strands. Therefore the fibrin deposited in the sample with IAM is not covalently cross-linked and gets disrupted upon urea treatment. In the sample without IAM the factor XIIIa does covalently cross-link the fibrin, presumably to bacterial surface proteins strengthening the fibrin/bacterium complex. The prolonged incubation time was necessary since the fibrinogen concentration used was approximately 100-fold lower than under physiological conditions to prevent the formation of a solid clot.

## Discussion

The role of fibrinogen in the human immune defense is not well explored but in some lower invertebrates like the horseshoe crab *Tachypleus tridentatus* a fibrinogen functional analogue, coagulogen, is regarded as a contributor to host defense. In *T. tridentatus* the clotting factors are found in hemocyte granules of which the contents are released on detection of LPS or peptidoglycan. The secretion leads, by two pathways, depending on the substance encountered, to the cleavage and activation of the proclotting enzyme generating the clotting enzyme. The clotting enzyme cleaves coagulogen, which afterwards can polymerize forming an insoluble gel aggregating the invading microorganisms [11].

There are many functional similarities between the horseshoe crab coagulation system and the mammalian since both are regulated by serpin type protease inhibitors, rely on cascade events to mount a powerful response and are mainly activated at the site of tissue damage [11], [16]. Additionally the proteins involved in the activation of clotting in *T. tridentatus* such as factor B (the protease which activates the proclotting enzyme if LPS is present), the proclotting enzyme and coagulogen appear to be functional analogues of factor X, prothrombin and fibrinogen, respectively, despite differences on the structural level. What sets the invertebrate and mammalian cascades apart is the knowledge of their roles as immune defense mechanisms. As mentioned above *T. tridentatus* use clotting to aggregate pathogens as well as maintaining hemostasis. In mammals the clotting system is mainly believed to be involved in maintaining hemostasis but recent reports also indicate that fibrin(ogen) and fibrinogen activation products might also play an important part in mediating clearance of pathogens. Upon activation of fibrinogen the fibrinopeptides A and B are released and these are chemotactic factors capable of recruiting neutrophils, macrophages and fibroblasts to the site of fibrinogen turnover [17]–[20]. Also fibrin(ogen) deposition in itself has been investigated for direct effects on pathogen neutralization. The role of fibrinogen has been investigated in *in vivo* studies using fibrinogen deficient mice or mice with modified fibrinogen, where binding of complement receptor 3 to fibrin(ogen) is prevented. These studies showed that fibrin(ogen) restrains the growth of *Listeria monocytogenes* in infected hepatic tissue and aids the bacterial clearance of *S. aureus* from the peritoneal cavity [21], [22]. Fibrin(ogen) has also been shown to increase adherence of phagocytes when biomaterials have been used as model for foreign bodies [23]. These observations indicate that fibrin(ogen) plays a role in the neutralization of invading pathogens since it mediates both migration and adhesion of phagocytes and in some cases, dependent on the pathogen, is capable of limiting growth as well as causing them to aggregate.

What we have reported here is a link between the complement system and the coagulation system. As the results show MASP2 is capable of generating thrombin via prothrombin. This activation is specific, in that MASP2 homologues do not activate prothrombin, and MASP2 does not activate other protease proenzymes. The activation occurs at physiological levels of prothrombin. The thrombin activation potential is quite low (about 4–5% the rate observed with factor Xa) and presumably much lower than would be observed for the prothrombinase complex. Since the prothrombinase complex mainly is generated on phospholipid membranes at the site of tissue damage a rapid and local thrombin generation is required to prevent loss of blood. In the case of MASP2 it will activate prothrombin near any surface where MBL or the ficolins can bind. Such surfaces do not have to be localized in one area but might be transported in the bloodstream round the body. If MASP2 was as efficient as the prothrombinase complex the activation of MASP2 could lead to thrombosis and therefore do more harm than good. Formation of a large clot around the MBL/ficolin-binding particle might well inhibit subsequent clearance by phagocytes as well. Instead MASP2 has a low activation potential but this ensures that only a limited amount of thrombin is generated and due to the surplus of inhibitors that are found in plasma it will only be active for a short time. This has two effects: firstly not much fibrin will be generated (probably not enough for forming a solid clot) and secondly the active thrombin will only remain active close to the site of activation since it most likely will be inhibited before it can diffuse far from the activation site. The limited, localized thrombin activity generated increases the chance of fibrin being deposited on the surface to which MASP2 is bound. This will result in the release of fibrinopeptides A and B and the deposition of fibrin that attracts phagocytes and serve as adhesion points for the immune system cells.

This activity might be a remnant activity of MASP2 from some ancestral protease. When C1s was tested we saw no ability to activate prothrombin, despite MASP2 and C1s being considered to be of common origin and being close homologues. It has previously been shown that MASP1 has thrombin-like activity, and activates factor XIII and cleaves fibrinogen directly [24]. When MBL or ficolins, with associated MASP1, 2 and 3 bind to targets (eg bacteria) MASP2 can subsequently activate the complement system through cleavage of C4 and C2. Both MASP1 and MASP2 may contribute to localized fibrinogen activation. The role of MASP3 remains unknown. We are currently studying the relative roles of MASP1 and MASP2 in fibrinogen and factor XIII activation.

Recently another study showed, like this one, that complement and coagulation might be more closely linked than previously believed. It was observed that C3 knock-out mice were capable of generating C5a. This activity was inhibitable by hirudin, a specific thrombin inhibitor, suggesting that this C5a generation was dependent on thrombin [25]. As our studies show, MBL/ficolin MASP2 complexes could contribute to the formation of thrombin required for the mechanism postulated in ref [25]. However incubation of C5 with thrombin does not produce significant cleavage of C5, when analyzed on SDS-PAGE (Krarup, unpublished observation) therefore one or more intermediate proteases might be required. Both our study and ref [25] suggest that the coagulation system and the complement system may be interconnected in several ways, reflecting their common origin and that they might be able to supplement each other to some extent in the case of deficiencies.

## Materials and Methods

### Biochemical methods

SDS-PAGE was done by the Laemmli system [26] or the Invitrogen NuPAGE® system (Invitrogen, Paisley, UK). Reducing sample buffer (0.2M Tris-HCl, 8M urea, 2% SDS, 2mM EDTA, pH 8.0) was prepared as described by Fairbanks et al (1971) [27].

Radio-iodination of fibrinogen was carried out using iodogen as catalyst [28] with 50 µg fibrinogen (Enzyme Research Laboratories Ltd, Swansea, UK) in 500 µl of 1.5 mM KH_2_PO_4_, 8.1 mM Na_2_HPO_4_, 140 mM NaCl, 2.7 mM KCl, 0.5 mM EDTA, pH 7.4 (PBS/EDTA) incubated 5 min on ice with 0.5 mCi Na^125^I (IMS30, GE Healthcare UK Limited, Chalfont St.Giles, UK). The specific activity was 5.7×10^6^ dpm/μg fibrinogen. The radioiodonated fibrinogen was diluted with unlabelled fibrinogen to achieve appropriate total fibrinogen concentration.

N-terminal sequencing was done by running protein samples on a 10% Novex Bis-Tris NuPAGE precast gel using MES buffer in a Novex×Cell II Mini-Cell gel apparatus. The protein bands were electroblotted onto a Novex 0.2 µm PVDF membrane (Invitrogen) using a Novex Blot module. The membrane was stained with Coomassie Brilliant Blue and target bands were excised. These were washed with 10% methanol and sequenced on a 494A Procise protein sequencer (Applied Biosystems, Warrington, UK) for 10 cycles using standard sequencing cycles [29].

### Incubation of prothrombin with activated trMASP2, trMASP1 or factor Xa

Prothrombin 3.3 µg (Haematologic Technologies Inc (HTI), Essex Junction, VT) was incubated with 30 ng factor Xa (HTI) or truncated rMASP2 (CCP1-CCP2-SP) [9] or trMASP1 (CCP1-CCP2-SP) [9] in 20 mM Hepes, 140 mM NaCl, 5 mM CaCl_2_, 0.05% Tween20 pH 7.4 (activation buffer) at 37°C in a total reaction volume of 30 µl. The samples were diluted 1:1 in reducing sample buffer and analyzed on SDS-PAGE.

### Incubation of prothrombin with other proteases

Prothrombin (2 µg) in a total volume of 30 µl was incubated as above alone or in the presence of 60 ng activated C1s, a mixture of activated C1s+C1r or factor Xa at 37°C. The C1s and C1r, C1s mixtures were purified as described in [30].

### Effect of factor Xa and MASP2 on VPR-AMC substrate turnover by prothrombin

To Microfluor^®^ white 96-well microtiter plate wells (Thermo Labsystems, Franklin, MA) were added 10 µg of prothrombin and 25 or 100 ng factor Xa or trMASP2, respectively. The thrombin substrate, Val-Pro-Arg-aminomethyl coumarin (VPR-AMC) (Bachem, Bubendorf, Switzerland) was added to a final concentration of 100 µM. The final volume/ well was 100 µl and reagents were all diluted in activation buffer. The samples were incubated for 1 hour at room temperature. The amount of cleaved substrate was continuously monitored and quantified in a microtiter plate reader (Fluoroscan, Thermo Life Sciences, Basingstoke, UK)

### Fibrinogen activation by rMBL and rMASP2 complexes

Rat rMBL-A [12] and full-length rMASP2K [13] were both expressed in the Chinese hamster ovary cell-line DXB11 using the pED vector [31]. The MASP2K has the arginine at the zymogen activation cleavage site replaced by a lysine residue. This yields an active form of MASP2 that is secreted as zymogen but is more stable than wild-type MASP2, since it only autoactivates in complex with MBL upon binding to a suitable surface [13]. The Arg to Lys mutation of MASP2K is not in the serine protease domain but in the linker region immediately preceeding it. The activated protease domains of wild-type MASP2 and MASP2K are thus identical. As expected, the MASP2K and wild-type MASP2 constructs cleave protein substrates (C2 or C4) with similar K_m_ and k_cat_ [9], [13], [32].

A 96-well Maxisorb microtiter plate (Nunc, Kamstrup, Denmark) was coated with 10 µg/ well mannan (Sigma-Aldrich, St.Louis, MO) in 0.1 M NaHCO_3_, pH 9.6 (coating buffer) overnight at 4°C. The volume added to each well was 100 µl unless otherwise stated. Residual binding sites were blocked with 200 µl of 1 mg/ml BSA in PBS/EDTA for 1h at room temperature. The wells were washed three times with activation buffer. Rat rMBL-A (0.12 µg) was preincubated in 100 µl activation buffer with or without 0.12 µg rat rMASP2K for 1 hour at 4°C. At the end of the incubation, sodium EDTA, pH 7.4 (final concentration 10 mM) was added to some samples to provide negative controls. Preincubated samples were then transferred to the mannan-coated wells, and left overnight at 4°C to allow the MBL/MASP2K complexes to bind to the mannan. The wells were then washed with activation buffer. Prothrombin (10 µg) and either fibrinogen (30 µg) or VPR-AMC (final concentration 100 µM) in 100 µl activation buffer was added to each well. The wells were incubated for at 37°C or room temperature, then analyzed either by SDS-PAGE to observe fibrinogen cleavage and polymerization, or in a fluorimeter to measure VPR-AMC turnover. Controls without prothrombin were also included. Fibrin clot formation was visualized by incubating a 2-fold dilution series of preincubated MBL/MASP2K complexes (starting at 0.2 µg) in 100 µl activation buffer in the mannan-coated microtiter plates as described above. Following the incubation the wells were washed three times with 200 µl activation buffer and incubated with 100 µl activation buffer with 10 µg prothrombin and 100 µg fibrinogen at 37°C. The light scatter at OD_405_ was used as a measure of fibrin polymerization as described in [33]. The % of maximum absorption was calculated by dividing the reading by that from wells incubated with 100 µg fibrinogen and 0.1 unit of human thrombin (Sigma-Aldrich).

### Fibrinogen deposition on mannan/ fibrinogen coated wells

Microtiter wells were coated with 100 µl of 5 µg/ml fibrinogen plus 5 µg/ml mannan in coating buffer overnight at 4°C. The wells were then blocked and washed as described above. Preformed rMBL/rMASP2K complexes (0.2 µg) were bound to the wells in 100 µl activation buffer and incubated overnight at 4°C. Controls with 10 mM EDTA, or with no MASP2K, were included. Wells were washed as before with activation buffer and 100 µl activation buffer with 10 µg prothrombin and 30 µg ^125^I-fibrinogen (30.000 cpm) added and incubated 37°C. An additional control without prothrombin was included. After the incubation wells were washed and the bound ^125^I-fibrin was quantified by measuring the whole well in a mini-assay type 6–20 gamma counter (Mini-instruments Ltd, Burnham-on-Crouch, UK).

### Fibrinogen deposition on Staphylococcus aureus derivatized beads

Dynabeads^®^ M-270 Amine (Invitrogen Ltd) derivatized with formalin-fixed whole *S. aureus* were prepared the following way. The *S. aureus* Wood strain (National Institutes of Health, Bethesda, MD) were grown and formalin fixed as described in [15] and beads and bacteria (approximately 1×10^9^ and 1×10^10^, respectively), were washed into 0.1 M sodium citrate, 140 mM NaCl, pH 9.5 in a total volume of 250 µl. The beads and the bacteria were mixed and sodium cyanoborohydride (Sigma-Aldrich) was added to a final concentration of 50 mM and incubated with stirring for 2 hours at room temperature. Residual active groups on the beads were blocked by 0.1 M ethanolamine pH 8.4 for 1 hour. After blocking the beads were washed and stored in activation buffer. MBL/MASP2K complexes were prepared as described above. *S. aureus* derivatized beads (2.5×10^7^) were mixed and incubated overnight at 4°C with 0.72 µg preformed MBL/MASP2K complexes in a total volume of 0.3 ml on a slow rotary stirrer. The beads were washed three times with 1 ml of activation buffer. The beads were resuspended in 0.5 ml activation buffer containing 50 µg prothrombin and ^125^I-fibrinogen (15 µg, 300000 cpm) and incubated at 37°C on a slow rotary stirrer. Controls with 1 mM Pefabloc SC (Pentapharm, Basle, Switzerland), 1 µg/ml human C1 inhibitor prepared as described in [34], [35] or 1 mM iodoacetamide (Sigma-Aldrich) were also prepared. After incubation the beads were washed and transferred to a new tube. The radioactivity associated with the beads was measured in the counter. The beads were resuspended and incubated in 6M urea (500 µl) for 30 min at room temperature with stirring. The radioactivity associated with the beads was measured as before.

### Specificity of prothrombin activation by MASP2

The potential cleavage and activation of other protease proenzymes namely plasminogen and prekallikrein by trMASP2 was tested as follows: Plasminogen (AB KABI, Stockholm, Sweden) and prekallikrein (Enzyme Research Laboratories Ltd.) (2 µg) were incubated with 20 ng trMASP2 or trMASP1 in 20 µl of activation buffer and for 1 hour or 16 hours at 37°C and analyzed on SDS-PAGE to determine whether the proenzymes had been cleaved. Formation of the active enzymes was assessed by incubating plasminogen or prekallikrein (1 µg) with 20 ng trMASP2 in a total volume of 200 µl activation buffer at room temperature with 100 µM Val-Leu-Lys-AMC or Phe-Pro-Arg-AMC, respectively, and the substrate turnover was measured for 2 hours. The two substrates are relatively specific for plasmin and kallikrein, respectively but are not cleaved by MASP2 [10].
